
JOURNAL INFORMATION
==============================
NLM Title Abbreviation: J Can Health Libr Assoc
Journal ID: J Can Health Libr Assoc
NLM Journal ID: 101229936
Journal ID: JCHLA

The Journal of the Canadian Health Libraries Association

EISSN: 1708-6892
Publisher: Canadian Health Libraries Association / Association des bibliotèques de la santé du Canada

ARTICLE INFORMATION
==============================
PMCID: PMC12352443
DOI: 10.29173/jchla29895
Article ID: JCHLA-46-075
Article version: 1
Subjects: Abstracts

CHLA 2025 CONFERENCE OTHER CONTENT / ABSC CONGRÈS 2025 AUTRES CONTENUS

Electronic publication date: 2025 Aug 1
Publication date: 2025 Aug
Volume: 46
Issue: 2
First page: 75
Last page: 76
Copyright: © Fournier
Copyright year: 2025
License: This article is distributed under a Creative Commons Attribution License: https://creativecommons.org/licenses/by/4.0/
License URL: https://creativecommons.org/licenses/by/4.0/

==============================
JOURNAL INFORMATION
==============================
NLM Title Abbreviation: J Can Health Libr Assoc
Journal ID: J Can Health Libr Assoc
NLM Journal ID: 101229936
Journal ID: JCHLA

The Journal of the Canadian Health Libraries Association

EISSN: 1708-6892
Publisher: Canadian Health Libraries Association / Association des bibliotèques de la santé du Canada

ARTICLE INFORMATION
==============================
Subjects: OC = Other Content

OC1. Advocacy and decision-making in health libraries

McKinnell Jennifer 1
Ross-White Amanda 2
Osborne Zachary 3
1 McMaster University,
2 Queen's University,
3 Unity Health Toronto

JOURNAL INFORMATION
==============================
NLM Title Abbreviation: J Can Health Libr Assoc
Journal ID: J Can Health Libr Assoc
NLM Journal ID: 101229936
Journal ID: JCHLA

The Journal of the Canadian Health Libraries Association

EISSN: 1708-6892
Publisher: Canadian Health Libraries Association / Association des bibliotèques de la santé du Canada

ARTICLE INFORMATION
==============================
Subjects: OC = Other Content

OC2. Birds of a feather: strategies, challenges, and lessons learned in proactive outreach in hospital libraries

Boghosian Talin
Ullah Sadaf
Mahood Quenby
Unity Health Toronto

